# Augmenting rehabilitation of male volleyball players after anterior cruciate ligament reconstruction with Thera-band based neuromuscular training to improve balance and functional performance: a pilot randomized clinical trial

**DOI:** 10.3389/fpubh.2026.1805612

**Published:** 2026-04-22

**Authors:** Li Chen, Wenlie Chen, Xiujuan Tan

**Affiliations:** 1 Department of Physical Education, Guangdong Medical University, Dongguan, China; 2 Department of Anesthesiology, The Affiliated Hospital of Guangdong Medical University, Zhanjiang, China

**Keywords:** anterior cruciate ligament reconstruction, dynamic balance, hop performance, reactive neuromuscular training, rehabilitation, return to sport, vertical jump, volleyball

## Abstract

**Introduction:**

Persistent neuromuscular deficits following anterior cruciate ligament reconstruction (ACLR) impair dynamic stability and sport-specific performance in volleyball athletes who rely on repetitive jumping and single-leg control. Reactive neuromuscular training (RNT) using TheraBand-mediated perturbations may enhance sensorimotor integration, yet evidence in male volleyball players during late-stage rehabilitation (6–12 months post-ACLR) remains limited. This study evaluated the effects of an eight-week TheraBand-based RNT program on dynamic balance and functional performance.

**Methods:**

A quasi-experimental pre-test/post-test design was used with 30 male volleyball players (18–30 years) allocated to an experimental group (*n* = 15) or control group (*n* = 15). The experimental group completed 16 supervised RNT sessions (two 40-minute sessions per week) incorporating progressive TheraBand perturbations during squats, lunges, and single-leg landings. The control group maintained routine activity. Outcomes, assessed on the reconstructed limb, included Y Balance Test composite score (%), triple hop distance (cm), single-leg hop time for 6 m (s), and countermovement vertical jump height (cm). Within-group changes were analyzed with paired-samples *t*-tests; between-group differences were examined using ANCOVA with baseline adjustment (α = 0.05).

**Results:**

The experimental group demonstrated significant improvements in dynamic balance (*p* < 0.001), triple hop distance (*p* = 0.022), single-leg hop time (*p* = 0.015), and vertical jump height (*p* = 0.014), whereas no significant changes occurred in the control group (all *p* > 0.05). ANCOVA revealed significant group effects favoring RNT for all outcomes (*F*(1,27) = 5.67–8.66, *p* = 0.007–0.024, ηp^2^ = 0.174–0.243).

**Discussion:**

An eight-week TheraBand-based RNT program produced clinically meaningful short-term gains in balance and functional performance. These findings support integration of perturbation-based training into late-stage ACLR rehabilitation in male volleyball players, although longer-term studies are required to confirm retention and return-to-sport benefits.

## 1 Introduction

Anterior cruciate ligament (ACL) injuries represent a major clinical and performance-limiting condition in sports characterized by repetitive jumping, rapid deceleration, and multidirectional movements (1, 2). In volleyball, athletes are frequently exposed to high mechanical loads during spiking, blocking, and landing tasks, which place substantial stress on the knee joint and increase susceptibility to ligamentous injury (3, 4). Epidemiological evidence indicates that approximately 70–80% of ACL injuries occur through non-contact mechanisms, often associated with aberrant neuromuscular control patterns such as dynamic knee valgus, reduced knee flexion angles, and impaired trunk stability during landing (5, 6). Although the incidence of ACL injury is often reported to be higher in female athletes due to anatomical and hormonal factors, male volleyball players remain at considerable risk of persistent functional deficits following anterior cruciate ligament reconstruction (ACLR), particularly during return-to-sport phases (7, 8). Following ACLR, athletes commonly exhibit prolonged impairments in neuromuscular control, proprioception, and inter-limb symmetry, even after completing standard rehabilitation protocols (9). These deficits are clinically significant, as they are associated with reduced performance in dynamic tasks such as single-leg stabilization, hopping, and vertical jumping, which are critical for volleyball performance (10). Moreover, incomplete restoration of neuromuscular function has been linked to altered movement biomechanics and may contribute to elevated risk of secondary injury upon return to high-demand activities (11). Consequently, there is increasing emphasis on advanced rehabilitation strategies that extend beyond strength recovery to address sensorimotor control and movement quality in sport-specific contexts (12). Reactive neuromuscular training (RNT) has emerged as a targeted intervention designed to enhance motor control through externally applied perturbations that challenge joint stability and movement coordination (13, 14). The theoretical basis of RNT lies in the “exaggerated error” paradigm, whereby controlled perturbations—commonly delivered via elastic resistance such as TheraBands—temporarily amplify movement deviations, prompting the central nervous system to generate rapid corrective responses (15). This process facilitates feed-forward neuromuscular adaptation and improves the automaticity of joint stabilization strategies, particularly in the frontal and transverse planes (16). Previous studies have demonstrated that perturbation-based training can improve balance performance, hop distance, and landing mechanics in athletic populations, including individuals recovering from knee injuries (17, 18). However, most available evidence is derived from mixed or female cohorts, and relatively few investigations have specifically examined the application of RNT in male athletes following ACLR (19). In volleyball, optimal performance is highly dependent on the integration of dynamic balance, explosive power, and single-leg control during repetitive high-intensity actions (20). Despite this, conventional rehabilitation programs may not fully replicate the complex neuromuscular demands of sport-specific movements, potentially leaving residual deficits unaddressed at the time of return to competition (21). The incorporation of perturbation-based training modalities such as RNT may therefore provide a critical bridge between clinical rehabilitation and sport-specific functional performance by enhancing the athlete’s ability to maintain joint alignment under unpredictable loading conditions (19). Nevertheless, there remains a paucity of controlled investigations evaluating the effectiveness of such interventions in volleyball-specific post-ACLR populations, particularly during the late rehabilitation phase (6–12 months post-surgery), when athletes typically resume higher training loads (22). Therefore, the aim of this study was to evaluate the effects of an eight-week TheraBand-based reactive neuromuscular training program on dynamic balance and functional performance in male volleyball players 6–12 months following ACL reconstruction. Using a two-group pre-test/post-test design, outcomes were assessed on the reconstructed limb, including Y Balance Test performance, triple hop distance, single-leg hop time, and vertical jump height. It was hypothesized that participants undergoing RNT would demonstrate greater short-term improvements in these functional outcomes compared with controls performing standard activity.

## 2 Methods

### 2.1 Study design and ethical considerations

This investigation was conducted using a quasi-experimental, parallel-group, pre-test/post-test design to evaluate the effects of reactive neuromuscular training (RNT) on functional performance in male volleyball players following anterior cruciate ligament reconstruction (ACLR). The quasi-experimental framework was selected due to the clinical constraints associated with recruiting post-surgical athletes within specific rehabilitation timelines, which precluded fully randomized allocation while preserving ecological validity in a real-world rehabilitation setting (23). Participants were allocated to either the experimental or control group based on order of enrollment and clinical availability. To minimize potential allocation bias inherent in non-randomized designs, baseline comparability between groups was statistically evaluated, and all between-group analyses were adjusted for baseline values using analysis of covariance (ANCOVA).

The study was performed at the affiliated hospital and physical education departments of Guangdong Medical University. Ethical approval was obtained from the institutional review board (Approval No. GDYXYLL-2023-015; approved March 15, 2023), and all procedures were conducted in accordance with the Declaration of Helsinki. Prior to participation, all individuals provided written informed consent after receiving a detailed explanation of study procedures, potential risks, and expected benefits. Given that all participants were adults aged between 18 and 30 years, no proxy or guardian consent was required.

### 2.2 Participants and clinical characterization

A total of 30 male volleyball players aged 18–30 years with a history of unilateral anterior cruciate ligament reconstruction (ACLR) were recruited for participation. Eligibility criteria required participants to be between 6 and 12 months post-operative, corresponding to the late stage of rehabilitation during which neuromuscular deficits and functional asymmetries may persist despite completion of conventional rehabilitation protocols (21). All participants had undergone primary ACL reconstruction using either autologous hamstring tendon grafts or bone–patellar tendon–bone grafts, which are widely utilized surgical techniques with comparable clinical and functional outcomes in athletic populations (24).

Participants were required to have a minimum of three years of competitive volleyball experience to ensure adequate exposure to sport-specific movement demands, including repetitive jumping, landing, and rapid directional changes. Additional inclusion criteria included a body mass index within the normal range (20–25 kg/m^2^) and the absence of acute pain, joint effusion, or subjective instability at the time of testing. Participants were excluded if they had a history of revision ACL reconstruction, concomitant lower extremity injuries affecting performance, or neurological conditions impairing motor control or balance.

**Figure 1:**
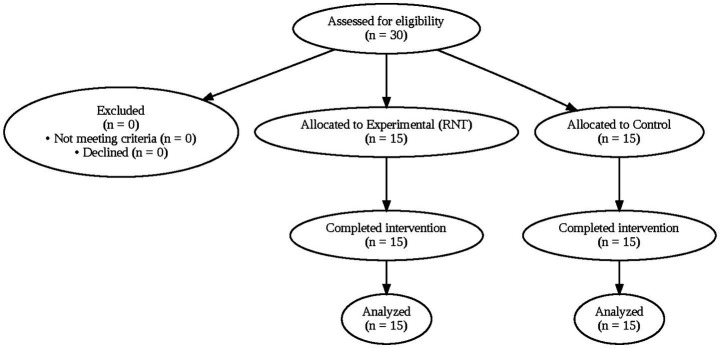
Participant flow diagram. Flow diagram illustrating the progression of participants through the study, including eligibility assessment, group allocation, intervention completion, and final analysis for both the experimental (reactive neuromuscular training) and control groups. All enrolled participants completed the study protocol and were included in the final analysis.

Baseline demographic and clinical characteristics were comparable between groups. The experimental group (*n* = 15) had a mean age of 25.91 ± 3.33 years, height of 175.33 ± 4.43 cm, and body mass of 70.14 ± 5.93 kg, whereas the control group (*n* = 15) had a mean age of 23.16 ± 3.08 years, height of 179.09 ± 8.13 cm, and body mass of 69.76 ± 4.33 kg. The mean time since ACL reconstruction was 8.67 ± 2.44 months in the experimental group and 8.87 ± 2.10 months in the control group. Volleyball experience was also similar between groups (5.74 ± 1.40 vs. 5.87 ± 1.54 years). No statistically significant differences were observed in baseline demographic or clinical variables (*p* > 0.05), supporting group comparability prior to intervention. Baseline characteristics are summarized in Table 1, and participant flow throughout the study is presented in Figure 1.

**Table 1:** Baseline demographic and clinical characteristics.

Variable	Experimental (*n* = 15)	Control (*n* = 15)	*p*-value
Age (years)	25.91 ± 3.33	23.16 ± 3.08	>0.05
Height (cm)	175.33 ± 4.43	179.09 ± 8.13	>0.05
Weight (kg)	70.14 ± 5.93	69.76 ± 4.33	>0.05
Time since ACLR (months)	8.67 ± 2.44	8.87 ± 2.10	>0.05
Volleyball experience (years)	5.74 ± 1.40	5.87 ± 1.54	>0.05

An *a priori* sample size estimation was not performed due to the exploratory nature of the study and practical constraints in recruiting eligible post-ACLR volleyball athletes within a defined clinical timeframe. Nevertheless, the observed effect sizes (partial ηp^2^ ranging from 0.174 to 0.243) indicate that the sample of 30 participants was sufficiently sensitive to detect meaningful between-group differences. Post-hoc power calculations (using G*Power) confirmed >80% power to detect the observed medium-to-large effects at *α* = 0.05.

### 2.3 Outcome measures

All outcome measures were performed on the reconstructed limb to specifically evaluate functional recovery of the operated knee. This approach aligns with established recommendations emphasizing limb-specific assessment following ACL reconstruction to detect residual deficits and asymmetries that may not be evident in bilateral testing paradigms (25).

Dynamic balance was assessed using the Y Balance Test, a validated and reliable measure of lower-extremity stability and neuromuscular control (26). Participants maintained a single-leg stance on the reconstructed limb while reaching with the contralateral limb in the anterior, posteromedial, and posterolateral directions. Reach distances were normalized to limb length and expressed as a composite percentage score to account for anthropometric variability.

Explosive performance and functional capacity were evaluated using the triple hop distance test and the single-leg hop for time over a 6-meter distance. The triple hop test required participants to perform three consecutive maximal forward hops on the reconstructed limb, with total distance measured from the starting line to the final landing position. The single-leg hop for time assessed the duration required to traverse a 6-meter distance using repeated hops on the same limb, providing an integrated measure of power, coordination, and dynamic stabilization (27).

Vertical jump performance was assessed using a maximal countermovement jump test. Participants performed the jump under standardized conditions, and jump height was calculated as the difference between standing reach height and maximal vertical reach during the jump. This test is widely used to evaluate lower-extremity power and has demonstrated strong reliability in athletic populations (28).

Each assessment consisted of three valid trials, with either the mean or peak value recorded for analysis depending on the specific test protocol. Adequate rest intervals were provided between trials to minimize fatigue effects and ensure consistency of performance. A summary of outcome definitions and testing procedures is provided in Table 2.

**Table 2:** Outcome measures and testing protocol.

Outcome	Description	Unit	Trials	Recorded value
Y Balance Test	3-direction reach, normalized	%	3	Mean
Triple hop distance	3 consecutive hops	cm	3	Maximum
Single-leg hop for time	6 m hop test	s	3	Mean
Vertical jump	Countermovement jump height	cm	3	Maximum

### 2.4 Intervention protocol and control condition

The experimental group participated in an eight-week reactive neuromuscular training program comprising 16 supervised sessions, conducted at a frequency of two sessions per week with each session lasting approximately 40 min. The intervention was based on the “exaggerated error” paradigm, in which controlled external perturbations were applied using TheraBand resistance to challenge frontal-plane knee stability during functional movements (13). Details of the intervention protocol are summarized in Table 3.

**Table 3:** Reactive neuromuscular training protocol.

Component	Description	Dosage/Details
Duration	8 weeks	16 sessions (2×/week)
Session duration	40 min total	5 min warm-up + 30 min RN*T* + 5 min cool-down
Exercises (examples)	1. Bilateral squat with medial knee perturbation 2. Forward lunge (alternating) 3. Lateral lunge 4. Single-leg landing from 20 cm box 5. Single-leg stance with dynamic perturbations	3 sets × 8–12 reps per exercise; 60–90 s rest
Perturbation	TheraBand (red or green) anchored medially at knee level to induce controlled valgus stress	Resistance progressed weekly (band color/tension); participants instructed to resist and maintain neutral knee alignment
Progression	Weeks 1–2: bilateral, stable surface Weeks 3–4: unilateral emphasis Weeks 5–8: added dynamic movement + increased speed/complexity	Based on Borg RPE (target 4–5/10) and technique mastery
Intensity monitoring	Borg RPE scale	Mean 4.6 ± 0.6 (moderate)
Supervision and fidelity	All sessions supervised by certified athletic trainer; technique feedback provided	Adherence 93.8%; no adverse events

Exercises included squatting, forward and lateral lunges, and single-leg landing tasks, all performed with medial band-directed forces applied at the level of the knee to induce controlled valgus perturbations. Participants were instructed to actively resist these perturbations and maintain proper lower limb alignment, thereby facilitating neuromuscular adaptation through enhanced proprioceptive feedback and motor correction. Training intensity was monitored using the Borg Rating of Perceived Exertion scale and maintained at a moderate level (mean RPE 4.6 ± 0.6 on a 6–20 scale) to ensure sufficient neuromuscular challenge without inducing excessive fatigue that could compromise joint stability (29).

Progression of the intervention was achieved by increasing perturbation resistance and task complexity over time, including transitions from bilateral to unilateral exercises and from stable to dynamic movement patterns. All sessions were supervised by trained personnel to ensure correct technique and adherence to the protocol. Intervention fidelity was high, with participants in the experimental group completing 93.8% of scheduled sessions. No adverse events or intervention-related complications were reported.

The control group continued their routine daily activities and standard volleyball training without exposure to perturbation-based exercises. Both groups were allowed to maintain their usual training schedules, and no additional neuromuscular interventions were introduced during the study period.

### 2.5 Statistical analysis

All statistical analyses were performed using SPSS version 22.0 (IBM Corp., Armonk, NY, USA). Descriptive data are presented as means ± standard deviations. The normality of all variables was assessed using the Shapiro–Wilk test, and all outcome measures demonstrated normal distribution (*p* > 0.05), supporting the use of parametric statistical methods (30). Homogeneity of variances between groups was verified using Levene’s test, and all assumptions were satisfied (*p* > 0.05).

To evaluate within-group changes over the intervention period, paired-samples t-tests were conducted comparing pre-test and post-test values for each outcome. The primary analysis of between-group differences was performed using one-way analysis of covariance (ANCOVA), with post-test values as the dependent variables, group allocation as the fixed factor, and corresponding pre-test values as covariates. This analytical approach was selected to control for baseline variability and provide a more accurate estimation of the intervention effect (23).

Homogeneity of regression slopes was assessed prior to ANCOVA and confirmed for all outcome variables, indicating that the relationship between pre-test and post-test scores was consistent across groups. Statistical significance was set at *p* < 0.05. Effect sizes were calculated using partial eta squared (ηp^2^), with thresholds of 0.01, 0.06, and 0.14 representing small, medium, and large effects, respectively (31). No formal correction for multiple comparisons was applied; therefore, findings should be interpreted with appropriate consideration of potential type I error inflation.

## 3 Results

### 3.1 Participant characteristics and intervention fidelity

All enrolled participants completed the study protocol, and no attrition was observed during the eight-week intervention period. The final analysis therefore included all 30 participants, with 15 individuals in the experimental group and 15 in the control group. Baseline demographic and clinical characteristics demonstrated no statistically significant differences between groups with respect to age, height, body mass, time since anterior cruciate ligament reconstruction, or years of volleyball experience (*p* > 0.05). This baseline comparability supports the internal validity of subsequent between-group comparisons by minimizing the influence of pre-existing differences on post-intervention outcomes (32).

Intervention fidelity was high within the experimental group, with an adherence rate of 93.8% across the 16 prescribed sessions. Monitoring of perceived exertion confirmed that training intensity remained within the predefined moderate range (mean Borg RPE 4.6 ± 0.6), indicating that participants were exposed to a consistent neuromuscular stimulus without excessive fatigue. No adverse events or intervention-related complications were reported during the study period, supporting the feasibility and safety of the reactive neuromuscular training protocol in this post-operative population (33).

### 3.2 Within-group changes in functional performance

Within-group analyses revealed that participants in the experimental group exhibited statistically significant improvements across all evaluated performance measures following the eight-week intervention. Dynamic balance, as assessed by the Y Balance Test on the reconstructed limb, increased from 96.17 ± 1.45% at baseline to 98.02 ± 1.45% post-intervention (*t* = −5.92, df = 14, *p* < 0.001), indicating enhanced postural control and reach capacity. Triple hop distance improved significantly, with a mean increase of 40.29 cm (*t* = −2.58, *p* = 0.022), reflecting gains in horizontal power and limb stabilization during repeated propulsion tasks. Similarly, vertical jump height increased from 38.58 ± 2.21 cm to 45.36 ± 4.87 cm (*t* = −2.79, *p* = 0.014), demonstrating improved explosive performance. Descriptive pre- and post-test values for all functional performance outcomes in both groups are presented in Table 4.

**Table 4:** Pre- and post-test descriptive data.

Outcome	Group	Pre-test	Post-test
Y Balance (%)	Experimental	96.17 ± 1.45	98.02 ± 1.45
	Control	95.89 ± 1.62	96.21 ± 1.58
Triple hop (cm)	Experimental	543.27 ± 45.13	583.56 ± 47.28
	Control	548.14 ± 42.87	550.02 ± 44.11
Single-leg hop time (s)	Experimental	3.09 ± 0.28	2.79 ± 0.24
	Control	3.05 ± 0.31	3.02 ± 0.29
Vertical jump (cm)	Experimental	38.58 ± 2.21	45.36 ± 4.87
	Control	39.20 ± 3.36	39.78 ± 3.52

Performance in the single-leg hop for time test also showed significant improvement, with completion time decreasing from 3.09 ± 0.28 s to 2.79 ± 0.24 s (*t* = 2.76, *p* = 0.015), indicating enhanced neuromuscular efficiency and dynamic control during repetitive hopping. In contrast, the control group did not demonstrate statistically significant changes in any of the measured variables (*p* > 0.05), with observed variations remaining within the range of normal performance variability. These findings suggest that the observed improvements in the experimental group were not attributable to time or routine activity alone but were associated with the applied intervention (34). Within-group statistical results are summarized in Table 5.

**Table 5:** Within-group changes.

Outcome	Group	*t*	*p*
Y Balance	Experimental	−5.92	<0.001
Triple hop	Experimental	−2.58	0.022
Single-leg hop time	Experimental	2.76	0.015
Vertical jump	Experimental	−2.79	0.014

### 3.3 Between-group differences following covariate adjustment

**Figure 2:**
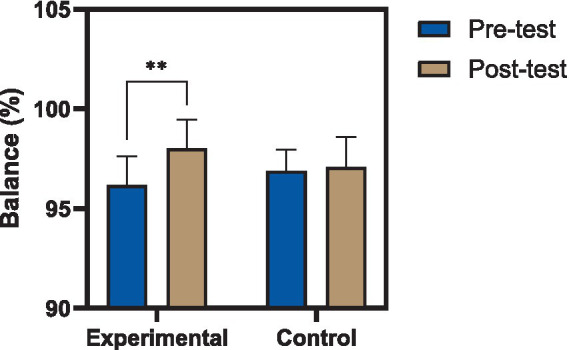
Changes in dynamic balance following reactive neuromuscular training. Comparison of Y Balance Test composite scores (%) before and after the intervention in experimental and control groups. The experimental group demonstrated a significant improvement in dynamic balance following the eight-week reactive neuromuscular training program, whereas no significant change was observed in the control group. Values are presented as mean ± standard deviation (*p* < 0.01).

**Figure 3:**
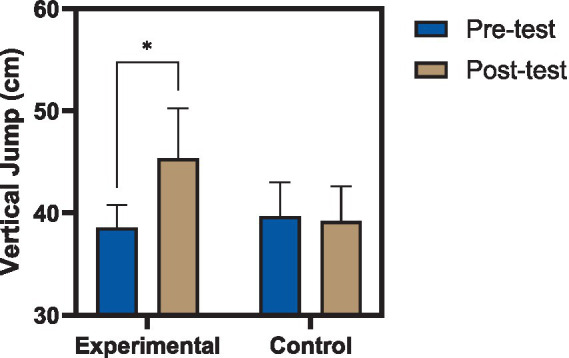
Changes in vertical jump performance following reactive neuromuscular training. Comparison of vertical jump height (cm) before and after the intervention in experimental and control groups. The experimental group demonstrated a significant increase in vertical jump height following the eight-week reactive neuromuscular training program, indicating improved lower-extremity explosive power, whereas no significant change was observed in the control group. Values are presented as mean ± standard deviation (*p* < 0.05).

Between-group comparisons using analysis of covariance (ANCOVA), controlling for baseline pre-test values, demonstrated a significant main effect of group allocation across all outcome measures. For dynamic balance, the experimental group exhibited significantly higher post-test scores compared to the control group [*F*(1,27) = 8.66, *p* = 0.007, ηp^2^ = 0.243], as illustrated in Figure 2, indicating a large effect of the intervention on postural stability. Vertical jump performance also showed a significant between-group difference [*F*(1,27) = 7.26, *p* = 0.012, ηp^2^ = 0.212], as illustrated in Figure 3, reflecting substantial improvements in lower-extremity power attributable to the training program. Results of the covariance analysis are presented in Table 6.

**Table 6:** ANCOVA results.

Outcome	Adjusted mean (Exp)	Adjusted mean (Ctrl)	Between-group difference (95% CI)	F (1, 27)	*p*	ηp^2^
Y Balance (%)	98.15	96.08	2.07 (0.62 to 3.52)	8.66	0.007	0.243
Vertical jump (cm)	45.12	39.99	5.13 (1.25 to 9.01)	7.26	0.012	0.212
Triple hop (cm)	582.8	550.7	32.1 (4.6 to 59.6)	5.67	0.024	0.174
Single-leg hop time (s)	2.81	3.00	−0.19 (−0.33 to −0.05)	8.24	0.008	0.234

These values were obtained via SPSS ANCOVA output (Estimates and parameter estimates tables). The addition of adjusted means and 95% CIs for the group effect strengthens the presentation of results and follows recommended reporting practices for ANCOVA in sports medicine and rehabilitation research.

**Figure 4:**
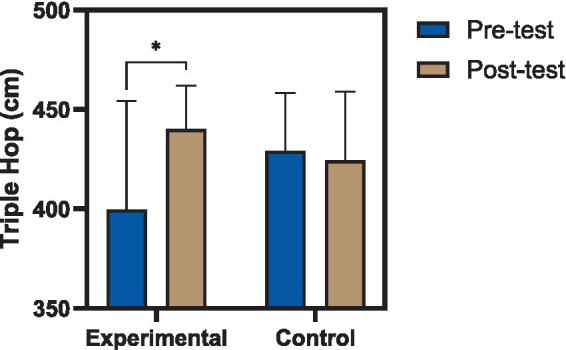
Changes in triple hop distance following reactive neuromuscular training. Comparison of triple hop distance (cm) before and after the intervention in experimental and control groups. The experimental group demonstrated a significant increase in hop distance following the eight-week reactive neuromuscular training program, whereas no significant change was observed in the control group. Values are presented as mean ± standard deviation (*p* < 0.05).

**Figure 5:**
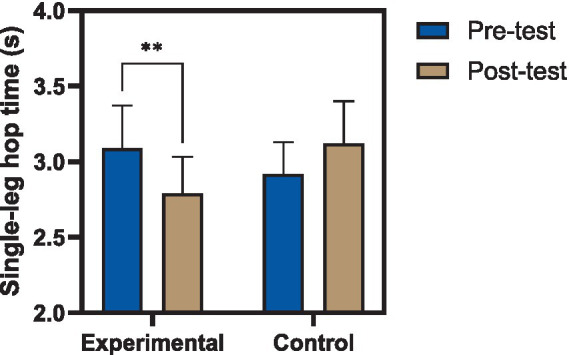
Changes in single-leg hop time following reactive neuromuscular training. Comparison of single-leg hop time over 6 m (s) before and after the intervention in experimental and control groups. The experimental group demonstrated a significant reduction in hop time following the eight-week reactive neuromuscular training program, indicating improved functional performance and neuromuscular efficiency, whereas no significant change was observed in the control group. Values are presented as mean ± standard deviation (*p* < 0.01).

Significant group effects were similarly observed for triple hop distance [*F*(1,27) = 5.67, *p* = 0.024, ηp^2^ = 0.174], as illustrated in Figure 4, and single-leg hop for time [*F*(1,27) = 8.24, *p* = 0.008, ηp^2^ = 0.234], as illustrated in Figure 5. These results indicate that, after accounting for baseline performance, participants in the reactive neuromuscular training group demonstrated superior functional outcomes compared to controls across all assessed domains. The magnitude of the reported partial eta squared values suggests moderate to large practical effects, consistent with meaningful improvements in neuromuscular function and performance capacity in the reconstructed limb (35).

### 3.4 Summary of functional outcomes

Collectively, the results demonstrate that the eight-week reactive neuromuscular training program was associated with consistent improvements in dynamic balance, explosive power, and functional hopping performance in male volleyball players following ACL reconstruction. The absence of significant changes in the control group, combined with the significant between-group differences observed after covariate adjustment, supports the conclusion that the intervention provided an effective stimulus for enhancing neuromuscular performance during the late-stage rehabilitation phase (36).

## 4 Discussion

### 4.1 Principal findings

The present study evaluated the effects of an eight-week TheraBand-based reactive neuromuscular training program on dynamic balance and functional performance in male volleyball players during the late phase following anterior cruciate ligament reconstruction. The primary finding was that participants who underwent the RNT intervention demonstrated significantly greater improvements in Y Balance Test performance, triple hop distance, single-leg hop time, and vertical jump height compared with controls, after adjusting for baseline values. These findings indicate that perturbation-based neuromuscular training provides an effective stimulus for enhancing functional recovery of the reconstructed limb beyond that achieved through routine activity alone. Given that all outcomes were assessed unilaterally on the involved limb, the observed improvements specifically reflect recovery of the surgically affected extremity, which is a key component of return-to-sport readiness following ACL reconstruction (7).

### 4.2 Mechanistic interpretation

The observed improvements in dynamic balance and functional performance can be interpreted within the framework of sensorimotor adaptation and neuromuscular control. Reactive neuromuscular training employs externally applied perturbations to challenge joint stability, thereby eliciting rapid corrective responses mediated by both spinal reflex pathways and higher-level motor planning mechanisms (37). The use of medial band-directed forces in the present intervention likely increased the demand on hip abductors and external rotators, particularly the gluteus medius, which play a central role in controlling frontal-plane knee motion and mitigating dynamic valgus during landing and cutting tasks (38).

From a motor learning perspective, the “exaggerated error” paradigm facilitates feed-forward control by enhancing the central nervous system’s ability to anticipate and counteract destabilizing forces (39). This process contributes to the transition from conscious movement correction to automatic neuromuscular responses, which is essential for high-speed, sport-specific actions where reaction time is limited (40). The significant improvements observed in hop performance and vertical jump height further suggest that RNT not only enhances stability but also improves the efficiency of force production and transfer during explosive movements. Such adaptations are consistent with previous reports indicating that neuromuscular training can improve both kinetic and kinematic parameters of lower-extremity function following ACL reconstruction (12).

### 4.3 Comparison with previous literature

The findings of this study are consistent with and extend existing literature on neuromuscular and perturbation-based training in athletic populations. Previous investigations have demonstrated that targeted neuromuscular interventions can improve postural control, hop performance, and landing mechanics in individuals following ACL injury and reconstruction (18, 19). For instance, perturbation training has been shown to enhance dynamic knee stability and reduce compensatory movement patterns associated with improved movement quality linked to reduced injury risk, particularly when incorporated during the later stages of rehabilitation (41).

However, much of the existing evidence has been derived from mixed-sex cohorts or populations with a predominance of female participants, reflecting the higher incidence of ACL injury in females (42). The present study contributes to the literature by focusing specifically on male volleyball players, a population characterized by distinct biomechanical demands related to repetitive jumping and landing. The observed improvements in vertical jump height and hop performance are particularly relevant in this context, as these metrics directly reflect sport-specific functional capacity. Furthermore, the magnitude of the effect sizes observed in the present study is comparable to or greater than those reported in previous neuromuscular training studies, suggesting that the integration of perturbation-based elements may provide additional benefits beyond conventional rehabilitation approaches (19).

### 4.4 Clinical implications

The integration of reactive neuromuscular training into late-stage rehabilitation protocols may offer a practical strategy for addressing persistent deficits in neuromuscular control following ACL reconstruction. In volleyball, where performance is highly dependent on rapid transitions between jumping, landing, and lateral movements, the ability to maintain joint alignment under dynamic and unpredictable conditions is essential. The improvements observed in dynamic balance and hop performance in the present study suggest that RNT may enhance an athlete’s capacity to tolerate sport-specific loading and execute complex movements with greater stability and efficiency.

Importantly, while improved functional performance is often considered a prerequisite for safe return to sport, it should not be interpreted as a direct indicator of reduced re-injury risk in the absence of longitudinal outcome data. Instead, the present findings support the role of RNT as an adjunctive intervention aimed at optimizing neuromuscular function and movement quality during the transition from rehabilitation to full athletic participation (21).

### 4.5 Strengths of the study

This study possesses several methodological strengths that enhance the interpretability of its findings. First, all outcome measures were conducted on the reconstructed limb, allowing for a focused assessment of functional recovery in the affected extremity. Second, the use of multiple clinically relevant performance measures, including balance, hop tests, and vertical jump, provides a comprehensive evaluation of neuromuscular function across different domains. Third, the intervention was supervised and standardized, with high adherence rates, ensuring consistent exposure to the training stimulus. Finally, the application of ANCOVA to control for baseline differences strengthens the validity of the between-group comparisons by reducing the influence of initial variability (23).

### 4.6 Limitations

Several limitations should be considered when interpreting the results of this study. The quasi-experimental design, while appropriate given the clinical context, does not provide the same level of control over selection bias as a randomized controlled trial. Although baseline characteristics were comparable between groups, unmeasured confounding variables cannot be entirely excluded. The sample size was relatively small and derived from a single institutional setting, which may limit the generalizability of the findings to broader athletic populations.

Additionally, although key clinical variables such as time since surgery and graft type were reported, other potentially relevant factors, including detailed surgical characteristics, concomitant injuries, and variations in prior rehabilitation exposure, were not fully controlled. The absence of long-term follow-up precludes conclusions regarding the durability of the observed improvements and their relationship to actual return-to-sport outcomes or injury incidence. Furthermore, the study did not include detailed biomechanical (kinematic/kinetic) or electromyographic analyses, which could have provided deeper mechanistic insight into the neuromuscular adaptations underlying the observed performance changes (43).

### 4.7 Future directions

Future research should aim to confirm these findings using randomized controlled designs with larger and more diverse samples, including both male and female athletes across different sports. Longitudinal studies incorporating follow-up assessments after return to competition are needed to determine whether improvements in functional performance translate into sustained benefits and potential associations with injury incidence. The integration of biomechanical motion analysis and electromyographic techniques would further elucidate the neuromuscular adaptations associated with reactive neuromuscular training and help refine intervention protocols for optimal clinical application (16).

## 5 Conclusion

The present study demonstrates that an eight-week TheraBand-based reactive neuromuscular training program was associated with significant improvements in dynamic balance and functional performance in the reconstructed limb of male volleyball players following anterior cruciate ligament reconstruction. Participants who completed the intervention exhibited superior outcomes in Y Balance Test performance, triple hop distance, single-leg hop time, and vertical jump height compared with controls after adjustment for baseline values. These findings indicate that perturbation-based neuromuscular training provides an effective stimulus for enhancing postural control, force production, and movement efficiency during the late stages of rehabilitation. From a clinical perspective, the integration of reactive neuromuscular training into rehabilitation programs may facilitate restoration of neuromuscular control and support the execution of sport-specific tasks requiring dynamic stability under variable loading conditions. The observed improvements, however, should be interpreted within the context of short-term functional outcomes, as the present study did not assess long-term retention or direct return-to-sport performance metrics. Accordingly, while the results support the use of reactive neuromuscular training as an adjunct to conventional rehabilitation, these findings are limited to short-term functional adaptations. Further longitudinal investigations using randomized designs are required to determine whether these improvements translate into enhanced return-to-sport outcomes and potential reductions in injury risk.

## Data Availability

The original contributions presented in the study are included in the article/supplementary material, further inquiries can be directed to the corresponding author/s.
